# Impact of COVID-19 on early identification of protein-energy malnutrition in the cancer care setting: a repeated cross-sectional survey of cancer care professionals

**DOI:** 10.1007/s00520-026-10338-1

**Published:** 2026-01-22

**Authors:** Marianne Boll Kristensen, Megan Crichton, Wolfgang Marx, Tateaki Naito, Egidio Del Fabbro, Elizabeth Isenring, Skye Marshall

**Affiliations:** 1 Department of Nutrition, Gødstrup Hospital, Herning, Denmark; 2 Cancer and Palliative Care Outcomes Centre, School of Nursing, Faculty of Health, Queensland University of Technology, Kelvin Grove, QLD Australia; 3 Deakin University, IMPACT (the Institute for Mental and Physical Health and Clinical Translation), Food & Mood Centre, Geelong, Australia; 4 School of Allied Health, Human Services and Sport, La Trobe University, Bundoora, Australia; 5 Cancer Supportive Care Center, Shizuoka Cancer Center, Nagaizumi, Shizuoka Japan; 6 Medical College of Georgia, Augusta University, Augusta, GA USA; 7 Bond University Nutrition and Dietetics Research Group, Faculty of Health Science & Medicine, Bond University, Robina, Queensland Australia; 8 Centre for Quality and Patient Safety Research, School of Nursing and Midwifery, Faculty of Health, Institute for Health Transformation, Deakin University, 1 Gheringhap St, Geelong, Victoria 3220 Australia

**Keywords:** Protein-energy malnutrition, Neoplasms, Health services, Nutrition screening, Nutritional assessment, COVID-19

## Abstract

**Purpose:**

What are the perceptions and practices regarding protein energy malnutrition (PEM) screening and assessment for patients with cancer among health professionals working in the cancer setting, and how have these perceptions and practices changed since the COVID-19 pandemic?

**Methods:**

A repeated cross-sectional study was conducted in 2018 (pre-COVID-19) and 2024 (post-COVID-19) using a study-specific online 24-item questionnaire. Qualified health professionals working as clinicians in the cancer setting were eligible. The survey was disseminated via professional associations internationally. Data were analysed in Stata/MP 18.5.

**Results:**

Of *N* = 282 participants included (*n* = 197 pre-COVID-19, *n* = 85 post-COVID-19), most were dietitians (39%) from Oceania (29%). The reporting of workplace nutrition screening policies increased between pre- and post-COVID-19, especially in North America (56% to 100%, *p* = 0.013). Barriers to nutrition screening remained consistent pre- and post-COVID-19 except in North America where limited awareness increased (50% to 93%, *p* = 0.019) and in Oceania where there was a decrease in incorrect use of a screening tool (63% to 36%, *p* = 0.027). Nutrition screening and assessment implementation remained mostly unchanged, except for an increase in patient self-screening (15% to 28%; *p* = 0.029) and fewer non-admission assessments conducted in Oceania.

**Conclusion:**

While some positive changes were observed between the pre- and post-COVID-19 period, nutrition screening and assessment continue to face many barriers. This has led to a lack of improvement or decline in nutrition screening and assessment practices in most regions and settings. PEM identification must evolve to ensure early detection for best patient care and in preparedness for future global health events.

**Supplementary Information:**

The online version contains supplementary material available at 10.1007/s00520-026-10338-1.

## Introduction

Globally, more than 19 million people are diagnosed with cancer every year [1], and 40%–80% will develop chronic disease-related protein-energy malnutrition (PEM) during their trajectory [2–4]. Chronic disease-related PEM is a complex, multifactorial condition characterised by unintentional loss of lean mass, with or without fat loss, in the presence of mild to moderate inflammation generated by chronic diseases such as cancer, arthritis, and organ failure [2, 5]. Also characterised by a loss of lean mass and physical function, sarcopenia may co-occur with PEM. The pathophysiological pathway for PEM in people with cancer, and its progression to cancer cachexia, involves inflammation caused by elevated pro-inflammatory cytokines. This leads to hypermetabolism, a prolonged acute phase protein response, increased muscle proteolysis, and impaired macronutrient metabolism [2, 6–9]. Other aetiological factors for all phenotypes of PEM include reduced dietary intake, impaired digestion and absorption of macronutrients, and/or excessive nutrient losses; all of which are highly influenced by societal, psychological, economic, and other personal circumstances of the individual [8]. Early identification of PEM is therefore critical for limiting its severe and widespread consequences, including greater risk of treatment toxicity [10], impaired physical function [11, 12], reduced quality of life [13, 14], and increased risk of death [15].

To identify patients at risk of PEM, international guidelines on nutrition in cancer consistently recommend that nutrition screening is performed at cancer diagnosis and repeated depending on the clinical situation [16–22]. PEM screening uses a nutrition screening tool that is quick and simple to implement. Screening tools may be implemented by the patient themselves, family, nurses, or other members of the healthcare team. However, the tool applied should be relevant to the patient group as demonstrated by validity and reliability studies [23, 24]. If a patient is identified as being at risk of PEM via a nutrition screening tool, a more thorough nutritional assessment should follow to determine if the patient meets diagnostic criteria for PEM, as well as determine the PEM phenotype with sufficient clinical information to inform a treatment plan [8, 16, 25]. Assessment for PEM should be completed via the application of a nutrition assessment tool validated for the patient group, and differs from a screening tool by being more comprehensive and applied by a specifically qualified and trained health professional [23, 24].

The implementation of such processes and tools may be hindered by the large variety of tools available, lack of or poorly implemented nutrition policies, and inadequate training and resourcing, which leads to confusion, inconsistency, de-prioritisation, incorrect application, inaction based on results, or use of clinical judgement rather than a validated tool [26–28]. Failure to address such barriers can lead to inadequate nutritional care of cancer patients and worsening PEM and/or cachectic states [29, 30]. Health care settings, researchers, and sector advocacy groups internationally have dedicated significant efforts to address these barriers and improve PEM identification and management, such as the Malnutrition Task Force [31], the Victorian Cancer Malnutrition Collaborative [32], and the Multinational Association of Supportive Care in Cancer (MASCC) Nutrition and Cachexia Study Group [33].

However, the COVID-19 pandemic caused substantial disruptions and stress to healthcare systems and professionals worldwide, including job burnout, workforce redistribution, new policies and procedures, and reorganisation of health system structures and resource allocations [34, 35]. In the cancer setting, there were delays and cancellations of cancer treatment, screening, and diagnosis, as well as a shift towards telemedicine without time or resources for appropriate implementation of systems to achieve optimal cancer care [36]. One Australian study conducted during the government-mandated lockdown in 2020 found adults diagnosed with cancer felt frustrated with a perceived prioritisation of COVID-19 over their cancer-related symptoms, and health care professionals reported facing additional challenges in maintaining quality cancer care [37]. With pre-existing barriers to PEM screening and assessment in cancer care, the long-term impact of the COVID-19 health system response on the identification of PEM is unknown. Research to meet this evidence gap can be used to assist PEM policies, research, and advocacy to appropriately adjust efforts in the post-COVID-19 era and facilitate implementation of best practice for the early identification of PEM in cancer settings.

### Research question

What are the perceptions and practices regarding PEM screening and assessment for patients with cancer among health professionals working in the cancer setting, and how have these perceptions and practices changed since the COVID-19 pandemic?

## Methods

### Study design and participants

This study was a repeated cross-sectional study approved by The La Trobe University Human Ethics Committee (HEC18133) with informed electronic consent. This study has been reported according to the Strengthening the Reporting of Observational Studies in Epidemiology (STROBE) Checklist: Cross-sectional Studies [38].

### Survey instrument

An online survey was conducted using Qualtrics software and was created and disseminated in English. A purpose-designed questionnaire was developed by a multidisciplinary project group consisting of experienced oncology clinicians and researchers from the MASCC Nutrition and Cachexia Study Group. The questionnaire consisted of 24 items about nutrition screening and assessment, one item about sarcopenia assessment, and one item about cancer cachexia assessment. Ten items determined participant characteristics, including demographics and professional experience. Items that addressed screening and assessment covered themes of perceptions of definitions, screening and assessment tools and practices, screening confidence, practice barriers, and policies. Items were a mixture of closed-ended, open-ended, and Likert scale questions. The questionnaire is available from the corresponding author upon reasonable request.

### Eligibility

Qualified health professionals working as clinicians, regardless of their discipline or country, were eligible to participate in the survey if they worked in the cancer setting. There were no exclusion criteria for this study.

### Data collection

The survey was implemented cross-sectionally from June 2018 to December 2018 (pre-COVID-19) and repeated from February 2024 to December 2024 (post-COVID-19). The survey was chosen to be repeated in 2024 to represent the post-COVID-19 era as there had been 1 to 2 years of post-pandemic healthcare ‘normalisation’, including introducing new health policies and adaptation to the ‘new normal’ [39] Distribution channels were chosen based on accessibility by the MASCC Nutrition and Cachexia Study Group core membership. All efforts were made to replicate the pre-COVID-19 survey distribution during the post-COVID-19 survey distribution; however, due to changes in policies and access of distribution channels, as well as a lower response rate in 2024, this could not be achieved (Box 1).

**Table:** **Box 1** Survey distribution channels for the implementation of the study-specific survey on nutrition screening and assessment perceptions and practices

Pre-COVID-19 survey distribution	Post-COVID-19 survey distribution
• Dietitians Association of Australia (now Dietitians Australia) • Australasian Society of Parenteral and Enteral Nutrition • Danish Dietetic Association • Danish Society of Cancer Nurses • Danish Society for Clinical Nutrition and Metabolism • Dietitian Connection • MASCC (full membership)	• Australasian Society of Parenteral and Enteral Nutrition • Clinical Oncology Society of Australia • Danish Dietetic Association • Danish Society of Cancer Nurses • Danish Society for Clinical Nutrition and Metabolism • Dietitian Connection • Levine Cancer Institute • LinkedIn (paid advertisement) • MASCC (Nutrition and Cachexia Study Group membership) • Professional networks of MASSC Nutrition and Cachexia Study Group core members • Queensland University of Technology School of Nursing

### Statistical analyses

All data were analysed in Stata/MP 18.5. Descriptive statistics were used. Frequencies were presented in numbers and percentages for all categorical variables. Comparisons between pre-COVID-19 and post-COVID-19 survey results for all participants were conducted using either the Chi-square test or Fisher’s exact test; the latter applied in cases where any cell had a count of < 5. For questions allowing multiple response options, tests were conducted for each individual response option by comparing the number of participants who selected the option to those who did not, acknowledging that the latter group may include participants who skipped the question entirely. For single-response questions, participants who did not select an answer were excluded from the statistical tests but retained in result tables and descriptive statistics to allow for assessment of potential non-response bias. A statistical significance level of 0.05 was applied. Due to the large number of statistical tests performed, statistically significant findings were interpreted with caution and clinical relevance was considered when drawing conclusions.

To assess whether observed differences between the pre- and post-COVID-19 survey results could be attributed to differences in sample composition, sensitivity analyses were performed on key items, adjusting for participants’ workplace(s). These analyses employed multinomial logistic regression for categorical outcomes and logistic regression for binary outcomes while differences were assessed using post-estimation Wald tests.

Only participants who stated that they conducted nutrition screening or nutrition assessment in their workplace were included in the analyses of practices regarding nutrition screening and assessment. For these analyses, in addition to comparisons between pre- and post-COVID-19 surveys for all participants, analyses were also conducted separately for each continent. Confidence in conducting nutrition screening and estimated time required to conduct a nutrition screening and nutrition assessment was assessed for dietitians, nurses, and physicians, and potential differences between subgroups were tested using Fisher’s Exact test.

## Results

### Participant characteristics

In total, *N* = 320 individuals gave consent to participate in the survey (*n* = 229 pre-COVID-19, *n* = 91 post-COVID-19). Thirty-two participants and *n* = 6 participants were excluded from the pre- and post-COVID-19 surveys, respectively, for not submitting any item responses. Therefore, a total of *N* = 282 participants were included for analysis (Table 1). Item response rate decreased from survey commencement in both groups, with the lowest response rates for the item on country of residence (72%). There were few participants from Asia to support interpretation of results (*n* = 18; Table 1).

**Table 1:** Characteristics of clinicians who responded to the study-specific survey on nutrition screening and assessment perceptions and practices

Characteristic	Pre-COVID-19 (*n* = 197)	Post-COVID-19 (*n* = 85)	Total (*N* = 282)
**Gender**, *n* (%)			
Female	116 (59)	49 (58)	165 (58)
Male	29 (15)	16 (19)	45 (16)
No response	52 (26)	20 (23)	72 (26)
**Profession**^**a**^, *n* (%)			
Dietitian	76 (39)	34 (40)	110 (39)
Nurse	27 (14)	9 (11)	36 (13)
Physician	37 (19)	20 (24)	57 (20)
Researcher	7 (4)	6 (7)	13 (5)
Other (dentist, pharmacist)	2 (1)	1 (2)	3 (1)
No response	53 (27)	20 (24)	73 (26)
**Highest qualification**, *n* (%)			
Bachelor’s degree	41 (21)	14 (16)	55 (19)
Postgraduate diploma	20 (10)	4 (5)	24 (9)
Master’s degree	47 (24)	28 (33)	75 (27)
Doctorate degree	36 (18)	19 (22)	55 (19)
No response	53 (27)	20 (24)	73 (26)
**Workplace**^**b**^, *n* (%)			
Hospital	124 (63)	38 (45)^e^	162 (57)
Outpatient/ambulatory	36 (18)	12 (14)	48 (17)
Hospice	1 (1)	1 (1)	2 (1)
University	24 (12)	5 (6)	29 (10)
Homecare	4 (2)	3 (4)	7 (2)
Private practice	7 (4)	3 (4)	10 (4)
Other	6 (3)	3 (3)	9 (3)
No response	52 (26)	20 (23)	72 (26)
**Years of oncology experience**, *n* (%)			
≤ 5 y	48 (24)	15 (18)	63 (22)
6–15 y	64 (33)	31 (36)	95 (34)
16–25 y	19 (10)	11 (13)	30 (11)
≥ 25 y	12 (6)	8 (9)	20 (7)
No response	54 (27)	20 (24)	74 (26)
**Work allocation in cancer care in past 12-months**, n (%)			
< 10%	16 (8)	8 (9)	24 (9)
10–30%	24 (12)	16 (19)	40 (14)
30–50%	24 (12)	8 (9)	32 (11)
> 50%	68 (35)	29 (34)	97 (34)
Not applicable^c^	11 (6)	4 (5)	15 (5)
No response	54 (27)	20 (24)	74 (26)
**Education in nutrition**^d^, *n* (%)			
Bachelor’s degree	74 (37)	43 (51)	117 (41)
Continuing education courses	13 (7)	4 (5)	17 (6)
Postgraduate courses	26 (13)	4 (5)	60 (11)
Own study	17 (9)	8 (9)	25 (9)
No education in nutrition	14 (7)	6 (7)	20 (7)
No response	53 (27)	20 (23)	73 (26)
**Place of residence**, *n* (%)			
Oceania	56 (28)	25 (29)	81 (29)
Europe	52 (26)	19 (22)	71 (25)
North America	18 (9)	14 (17)	32 (11)
South America	2 (1)	0	2 (1)
Asia	11 (6)	7 (8)	18 (6)
No response	58 (30)	20 (24)	78 (28)

For single-response items, rounded percentages may have been adjusted by ± 1 percentage point to ensure a total of 100%, with adjustments based on proximity to rounding thresholds

^a^Participants could provide more than one response to this question

^b^In the pre-COVID-19 survey, participants could provide more than one answer to this question. In the post-COVID-19 survey, only one answer was allowed

^c^Not applicable as participant does not see patients clinically

^d^Had nutrition content included as part of their medical or professional education

^e^Statistically significant difference between pre-COVID-19 and post-COVID-19 groups (*p* = 0.004, Chi-square test)

Participants most frequently reported being dietitians (39%), based in Oceania (29%) or Europe (25%) and having ≥ 6 years of oncology experience (52%). The only statistically significant difference between groups was a larger proportion of participants working in hospitals pre-COVID-19 compared to post-COVID-19 (63% vs. 45%, *p* = 0.004) (Table 1). Participants could provide more than one answer to the question regarding profession, and those who did, selected ‘Researcher’ (5%) in addition to a health professional discipline. In total, participants represented 25 countries across surveys, with no significant difference between pre- and post-COVID-19 surveys (*p* = 0.139; data not shown).

### Pre- and post-COVID-19 perceptions and practices regarding protein-energy malnutrition, cancer cachexia, and sarcopenia identification in the cancer setting

Both before and after COVID-19, PEM was most frequently perceived as undernutrition (pre-COVID-19, 51%; post-COVID-19, 68%) (Table 2). Prior to COVID-19, the most perceived challenges when providing nutritional care for patients with cancer were inconsistent follow-up (48%), financial barriers (44%), and health system-related barriers (43%). These remained the top three challenges post-COVID-19, with health system-related barriers increasing to 57% (*p* = 0.040), although a significant increase was also seen in language barriers from 10% to 22% (*p* = 0.006) (Table 2). Across continents, there was little variation, with at least two of these top three challenges consistently among the four most frequently reported challenges both before and after COVID-19 (Table 2).

**Table 2:** Pre- and post-COVID-19 perceptions and practices of clinicians working in the cancer setting regarding protein-energy malnutrition in the cancer setting according to geographic region^a^

	All participants		Oceania		Europe		North America		Asia	
	Pre-COVID-19 (*n* = 197)	Post-COVID-19 (*n* = 85)	Pre-COVID-19 (*n* = 56)	Post-COVID-19 (*n* = 25)	Pre-COVID-19 (*n* = 52)	Post-COVID-19 (*n* = 19)	Pre-COVID-19 (*n* = 18)	Post-COVID-19 (*n* = 14)	Pre-COVID-19 (*n* = 11)	Post-COVID-19 (*n* = 7)
**What ‘malnutrition’ was perceived to mean in the clinical context**, *n* (%)										
Undernutrition	118 (60)	58 (68)	39 (70)	17 (68)	31 (60)	11 (58)	9 (50)	9 (64)	8 (73)	6 (86)
Overnutrition	0	0	0	0	0	0	0	0	0	0
Both undernutrition and overnutrition	75 (38)	24 (28)	16 (28)	7 (28)	20 (38)	7 (37)	9 (50)	5 (36)	3 (27)	1 (14)
Other	2 (1)	2 (3)	1 (2)	1 (4)	1 (2)	1 (5)	0	0	0	0
No response	2 (1)	1 (1)	0	0	0	0	0	0	0	0
**Perceived common nutritional challenges faced when caring for patients with cancer**^**b**^, *n* (%)										
Inconsistent follow up	94 (48)	39 (46)	27 (48)	12 (48)	30 (58)	12 (63)	12 (67)	6 (43)	5 (45)	2 (29)
Health systems related barriers	85 (43)	48 (57)^d^	28 (50)	14 (56)	26 (50)	10 (53)	10 (56)	10 (71)	3 (27)	3 (43)
Financial	87 (44)	44 (52)	38 (68)	15 (60)	7 (13)	4 (21)	10 (56)	11 (79)	8 (73)	4 (57)
Health Literacy	76 (39)	35 (41)	32 (57)	12 (48)	12 (23)	5 (26)	9 (50)	7 (50)	7 (64)	1 (14)^k^
Limited access to allied health	56 (28)	26 (31)	18 (32)	8 (32)	11 (21)	5 (26)	7 (39)	7 (50)	3 (27)	2 (29)
Cultural barriers	36 (18)	19 (22)	18 (32)	6 (24)	3 (6)	3 (16)	2 (11)	5 (36)	7 (64)	2 (29)
Geographical considerations	33 (17)	14 (16)	19 (34)	4 (16)	3 (6)	3 (16)	2 (11)	3 (21)	1 (9)	0
Language barriers	20 (10)	19 (22)^e^	14 (25)	5 (20)	0	2 (11)^l^	2 (11)	4 (29)	1 (9)	1 (14)
Other	25 (13)	12 (14)	4 (7)	3 (12)	11 (21)	3 (16)	1 (6)	2 (14)	2 (18)	0
No response	5 (3)	0	0	0	0	0	0	0	0	0
**Malnutrition identification practices utilised in participants’ workplaces**, *n* (%)										
Nutrition screening	18 (9)	12 (14)	1 (2)	4 (16)^f^	10 (19)	4 (21)	2 (11)	0	2 (18)	2 (29)
Nutrition assessment	11 (6)	2 (2)	0	2 (8)^f^	4 (8)	0	3 (17)	0	3 (28)	0
Both nutrition screening and assessment	126 (64)	56 (66)	55 (98)	18 (72)^f^	36 (69)	15 (79)	12 (67)	14 (100)	3 (27)	3 (43)
Neither	8 (4)	6 (7)	0	1 (4)^f^	2 (4)	0	1 (5)	0	3 (27)	2 (28)
No response	34 (17)	9 (11)	0	0	0	0	0	0	0	0
**Sarcopenia assessed in participants’ workplace**, *n* (%)										
Yes	80 (41)	31 (36)	18 (32)	6 (24)	22 (42)	7 (37)	12 (67)	8 (57)	2 (18)	2 (29)
No	97 (49)	44 (52)	36 (64)	16 (64)	23 (44)	10 (53)	4 (22)	4 (29)	9 (82)	5 (71)
Unsure	19 (10)	9 (11)	2 (4)	3 (12)	7 (14)	2 (11)	2 (10)	2 (14)	0	0
No response	1 (0)	1 (1)	0	0	0	0	0	0	0	0
**Cachexia assessed in participants’ workplace**, *n* (%)										
Yes	104 (53)	43 (51)	32 (57)	11 (44)	31 (60)	7 (37)	11 (61)	12 (86)	6 (55)	4 (57)
No	61 (31)	27 (32)	22 (39)	12 (48)	14 (27)	7 (37)	3 (17)	2 (14)	5 (45)	3 (43)
Unsure	22 (11)	8 (9)	2 (4)	2 (8)	7 (13)	5 (26)	4 (22)	0	0	0
No response	10 (5)	7 (8)	0	0	0	0	0	0	0	0
**Existing nutrition screening policy at participants’ workplace**, *n* (%)										
Yes	128 (65)	60 (71)	51 (91)	19 (76)	40 (77)	17 (90)	10 (55)	14 (100)^g^	5 (46)	5 (71)
No	25 (13)	7 (8)	3 (5)	2 (8)	10 (19)	1 (5)	5 (28)	0^ g^	5 (45)	2 (29)
Unsure	10 (5)	5 (6)	2 (4)	4 (16)	2 (4)	1 (5)	3 (17)	0^ g^	1 (9)	0
No response	34 (17)	13 (15)	0	0	0	0	0	0	0	0
**Perceived barriers to successful implementation of nutrition screening in participants’ workplace**^c^, *n* (%)										
Insufficient time	91 (46)	41 (48)	34 (61)	16 (64)	33 (63)	14 (74)	5 (28)	4 (29)	10 (91)	5 (71)
Limited awareness regarding the importance of nutrition screening	87 (44)	41 (48)	39 (70)	12 (48)^m^	27 (52)	11 (58)	9 (50)	13 (93)^h^	5 (45)	4 (57)
Insufficient staff	66 (34)	40 (47)^i^	20 (36)	13 (52)	23 (44)	13 (68)^n^	7 (39)	6 (43)	9 (82)	7 (100)
Incorrect use of screening tool	56 (28)	18 (21)	35 (63)	9 (36) ^j^	12 (23)	4 (21)	5 (28)	5 (36)	2 (18)	0
High patient turnover	51 (26)	18 (21)	19 (34)	7 (28)	19 (37)	6 (32)	3 (17)	2 (14)	6 (55)	3 (43)
Unclear responsibility	35 (18)	18 (21)	13 (23)	8 (32)	13 (25)	6 (32)	1 (6)	2 (14)	3 (27)	2 (29)
Insufficient funding	18 (9)	12 (14)	8 (14)	6 (24)	4 (8)	1 (5)	3 (17)	2 (14)	3 (27)	3 (43)
Social barriers	7 (4)	4 (5)	2 (4)	0	1 (2)	1 (5)	2 (11)	2 (14)	0	0
No response	49 (25)	17 (20)	1 (2)	0	1 (2)	0	2 (11)	0	0	0

For single-response items, rounded percentages may have been adjusted by ± 1 percentage point to ensure a total of 100%, with adjustments based on proximity to rounding thresholds

^a^Data from South America are not shown, since only two participants pre-COVID-19 were from South America, and none post-COVID-19

^b^Participants could provide more than one answer

^c^Participants were asked to indicate what they perceived as the top three most pertinent barriers

^d–j^Statistically significant differences between groups (pre-COVID-19 vs. post-COVID-19): ^d^*p* = 0.040 (Chi-square test), ^e^*p* = 0.006 (Chi-square test), ^f^*p* = 0.001 (Fisher’s exact test), ^g^*p* = 0.013 (Fisher’s exact test), ^h^*p* = 0.019 (Fisher’s exact test), ^i^*p* = 0.031 (Chi-square test), ^j^*p* = 0.027 (Chi-square test)

^k–n^Trends indicating possible differences between groups (pre-COVID-19 vs. post-COVID-19) with *p*-values > 0.05 and ≤ 0.10): ^k^*p* = 0.066 (Fisher’s exact test), ^l^*p* = 0.069 (Fisher’s exact test), ^m^*p* = 0.062 (Chi-square test), ^n^*p* = 0.071 (Chi-square test)

Both nutrition screening and assessment were reported to be used to identify PEM in the workplace pre-COVID-19 (64%), and this remained unchanged post-COVID-19 (66%). This pattern was seen across most continents. However, in North America, 100% of participants post-COVID-19 reported using both methods, and in Europe and Oceania, the combined use remained consistently high, though a decline was noted in Oceania (98% to 72%, *p* = 0.001). Most participants reported that cancer cachexia was assessed at their workplace both pre- (53%) and post-COVID-19 (51%). Assessment of sarcopenia remained less frequent and largely unchanged (41% to 36%), with considerable variation across continents (Table 2).

Existing nutrition screening policies were reported in 65% of workplaces pre-COVID-19 and in 71% post-COVID-19. These policies were especially prevalent in Oceania pre-COVID-19 (91%) and North America, where the proportion increased from 56% to 100% post-COVID-19 (*p* = 0.013) (Table 2).

The most pertinent perceived barriers to implementation of nutrition screening prior to COVID-19 were insufficient time (46%), limited awareness regarding the importance of nutrition screening (44%), and insufficient staff (34%). These three barriers were still the most pertinent perceived after COVID-19, where the proportion of participants who reported insufficient staff as a barrier had increased to 47% (*p* = 0.031) (Table 2). Statistically significant changes within continents included an increase in the proportion of North American participants reporting limited awareness as a barrier (50% to 93%, *p* = 0.019) and a decrease in the proportion of Oceanian participants reporting incorrect use of screening tool as a barrier (63% to 36%, *p* = 0.027) (Table 2). Sensitivity analyses, adjusting for participants’ workplace(s), did not alter the overall results (Supplementary File 1).

### Nutrition screening practices of participants who work in the clinical cancer care setting

Within the subsample of participants who reported using nutrition screening in a clinical cancer setting, specific screening tools were routinely used by 83% pre-COVID-19, least often in North America (57%) and most often in Oceania (98%) (Table 3). While the Malnutrition Screening Tool (MST) was used most often overall (44%), it was only used by 2% of participants in Europe. Nurses were reported to contribute to nutrition screening the most often in Oceania and Europe (72% and 96%), whereas in North America, dietitians/nutritionists and physicians were most often involved in nutrition screening (43% for both). Nutrition screening was predominantly conducted at admission (40%) or within 24 h of admission (47%), which was consistent across all continents. Monitoring practices varied considerably.

**Table 3:** Nutrition screening methods used by participants who responded that they use nutrition screening in their cancer care workplace

	All participants		Continent^a^					
			Oceania		Europe		North America	
	Pre-COVID-19 (*n* = 144)	Post-COVID-19 (*n* = 68)	Pre-COVID-19 (*n* = 56)	Post-COVID-19 (*n* = 22)	Pre-COVID-19 (*n* = 46)	Post-COVID-19 (*n* = 19)	Pre-COVID-19 (*n* = 14)	Post-COVID-19 (*n* = 14)
**Method used to conduct nutrition screening in the clinical cancer care setting**^**b**^, *n* (%)								
Routine use of a specific screening tool	119 (83)	53 (78)	55 (98)	19 (86)^k^	37 (80)	16 (84)	8 (57)	12 (86)
Other^c^	78 (54)	36 (53)	26 (46)	11 (50)	26 (57)	11 (58)	9 (64)	6 (43)
**Nutrition screening tool used the most often**, *n* (%)								
Scored PG-SGA Short Form	14 (10)	7 (10)^d^	3 (5)	1 (5)^e^	4 (9)	1 (5)	4 (29)	2 (14)
MUST	12 (8)	2 (3)^d^	5 (9)	0^e^	7 (15)	2 (11)	0	0
MST	64 (44)	24 (35)^d^	46 (82)	15 (68)^e^	1 (2)	0	7 (50)	7 (50)
SNAQ	7 (5)	0 ^d^	0	0^e^	4 (9)	0	0	0
SGA	2 (1)	0 ^d^	1 (2)	0^e^	0	0	0	0
NRS 2002	10 (7)	9 (13)^d^	0	0^e^	10 (22)	9 (47)	0	0
MNA Short Form	4 (3)	6 (9)^d^	1 (2)	1 (4)^e^	1 (2)	3 (16)	0	0
Other tools	0	6 (9)^d^	0	2 (9)^e^	0	1 (5)	0	2 (14)
No standardised tool	14 (10)	12 (18)^d^	0	3 (14)^e^	6 (13)	3 (16)	3 (21)	3 (22)
No response	17 (12)	2 (3)	0	0	13 (28)	0	0	0
**Members of the participants team who contribute to nutrition screening**^**b**^, *n* (%)								
Patient (self-screening)	22 (15)	19 (28)^f^	4 (7)	5 (23)	10 (22)	3 (16)	5 (36)	7 (50)
Physician	29 (20)	14 (21)	4 (7)	2 (9)	13 (28)	6 (32)	6 (43)	2 (14)
Nurse	109 (76)	43 (63)^l^	54 (96)	16 (73)^g^	33 (72)	14 (74)	4 (29)	7 (50)
Dietitian/Nutritionist	69 (48)	39 (57)	25 (45)	10 (45)	22 (48)	14 (74) ^m^	6 (43)	8 (57)
Nutrition Assistant	28 (19)	11 (16)	19 (34)	7 (32)	4 (9)	3 (16)	1 (7)	0
No standardised nutrition screening	2 (1)	5 (7)^h^	0	1 (5)	1 (2)	1 (5)	1 (7)	2 (14)
No response	5 (3)	2 (3)	0	0	1 (2)	0	2 (17)	0
**When nutrition screening is conducted**^**b**^, *n* (%)								
At admission	58 (40)	28 (41)	21 (38)	9 (41)	20 (43)	8 (42)	6 (43)	8 (57)
Within 24 h of admission	67 (47)	32 (47)	32 (57)	12 (55)	20 (43)	9 (47)	5 (36)	7 (50)
Within 48 h of admission	23 (16)	10 (15)	8 (14)	1 (5)	9 (20)	6 (32)	2 (14)	1 (7)
Within 72 h of admission	6 (4)	4 (6)	1 (2)	1 (5)	1 (2)	0	1 (7)	1 (7)
No response	8 (6)	2 (3)	1 (2)	0	5 (11)	0	1 (7)	0
**Frequency of monitoring nutritional status and/or risk factors**^**b**^, *n* (%)								
Never	17 (12)	5 (7)	5 (9)	0	8 (17)	2 (11)	1 (7)	0
Weekly as inpatients	74 (51)	30 (44)	39 (70)	16 (73)	24 (52)	9 (47)	3 (21)	4 (29)
Every outpatient appointment	45 (31)	37 (54) ^i^	23 (41)	15 (68) ^j^	10 (22)	6 (32)	7 (50)	10 (71)
At discharge	13 (9)	8 (12)	9 (16)	4 (18)	2 (4)	0	1 (7)	4 (29)
Other	31 (22)	10 (15)	8 (14)	1 (5)	12 (26)	5 (26)	5 (36)	2 (14)
No response	8 (6)	2 (3)	0	0	1 (2)	0	0	0

*MNA* Mini Nutritional Assessment, *MST* Malnutrition Screening Tool, *MUST* Malnutrition Universal Screening Tool, *NRS* Nutrition Risk Screening, *PG-SGA* Patient-Generated Subjective Global Assessment, *SGA* Subjective Global Assessment, *SNAQ* Short Nutritional Assessment Questionnaire

For single-response items, rounded percentages may have been adjusted by ± 1 percentage point to ensure a total of 100%, with adjustments based on proximity to rounding thresholds

^a^Due to the low number of participants from Asia reporting use of nutrition screening in their workplace (*n* = 5 pre-COVID-19, *n* = 5 post-COVID-19), these data were not presented. Similarly, data from South America were not shown, since only two participants pre-COVID-19 were from South America, and none post-COVID-19

^b^Participants could provide more than one answer

^c^Other methods included screening by cancer site/stage, current body weight, or body mass index

^d–j^Statistically significant differences between groups (pre-COVID-19 vs. post-COVID-19): ^d^*p* = 0.001 (Fisher’s exact test), ^e^*p* = 0.008 (Fisher’s exact test), ^f^*p* = 0.029 (Chi-square test), ^g^*p* = 0.005 (Fisher’s exact test), ^h^*p* = 0.036 (Fisher’s exact test), ^i^*p* = 0.001 (Chi-square test), ^j^*p* = 0.031 (Chi-square test)

^k–m^Trends indicating possible differences between groups (pre-COVID-19 vs. post-COVID-19) with *p*-values > 0.05 and ≤ 0.10: ^k^*p* = 0.066 (Fisher’s exact test), ^l^*p* = 0.060 (Chi-square test), ^m^*p* = 0.056 (Chi-square test)

Post-COVID-19, the routine use of specific screening tools remained stable (83% to 78%; *p* > 0.05). The MST remained the most used tool overall (35%), but the overall distribution of responses regarding the most frequently used tool changed significantly (*p* = 0.001), possibly due to an increase in participants reporting no standardised tool (10% to 18%). In terms of who was involved in nutrition screening, a greater proportion of participants reported patients self-screening post-COVID-19 (from 15% to 28%), particularly in Oceania (7% to 23%) and North America (36% to 50%). There was a rise in participants reporting routine monitoring of nutrition status at every outpatient appointment (from 31% to 54%, *p* = 0.001), with the largest and only significant increase seen in Oceania (41% to 68%, *p* = 0.031).

### Nutrition assessment practices of participants who work in the clinical cancer care setting

Among the subsample of participants who reported using nutrition assessment in a clinical cancer setting, dietitians were most frequently reported to conduct the nutrition assessment pre-COVID-19 (72%), least frequently in North America (60%), and most frequently in Oceania (95%). The Scored PG-SGA was the most commonly used nutrition assessment tool overall (20%) (Table 4). Among the overall assessment methods, anthropometry and functional assessment were most used, while body composition analysis and pathology were least frequently reported. Consistent across continents, nutrition assessment was most frequently conducted when a patient was identified as at nutritional risk (58–96%). Following COVID-19, overall practices remained largely consistent. Dietitians continued to be the primary professionals conducting nutritional assessments (74%), and the Scored PG-SGA remained the most widely used tool (40%). Despite a small increase in patient self-assessment in Oceania (5% to 15%), the practice decreased significantly in Europe (from 28 to 0%, *p* = 0.025) and was no longer reported in North America.

**Table 4:** Nutrition assessment methods used by participants who responded that they use nutrition assessment in their cancer care workplace

	All participants		Continent^a^					
			Oceania		Europe		North America	
	Pre-COVID-19 (*n* = 137)	Post-COVID-19 (*n* = 58)	Pre-COVID-19 (*n* = 55)	Post-COVID-19 (*n* = 20)	Pre-COVID-19 (*n* = 40)	Post-COVID-19 (*n* = 15)	Pre-COVID-19 (*n* = 15)	Post-COVID-19 (*n* = 14)
**Nutrition assessment tool used the most often**, *n* (%)								
MUST	7 (5)	2 (3)^m^	0	1 (5)^n^	5 (13)	0	0	0
MNA	5 (4)	3 (5)^m^	0	1 (5)^n^	1 (2)	2 (13)	3 (20)	0
Scored PG-SGA	40 (29)	23 (40)^m^	27 (49)	11 (55)^n^	5 (13)	4 (27)	3 (20)	5 (36)
SGA	27 (20)	4 (7)^m^	21 (38)	4 (20)^n^	2 (4)	0	2 (13)	0
Other	27 (20)	17 (29)^m^	4 (7)	3 (15)^n^	19 (48)	8 (53)	2 (13)	5 (36)
No response	31 (22)	9 (16)	3 (6)	0	8 (20)	1 (7)	5 (34)	4 (28)
**Members of the participants team who contribute to nutrition assessment**^**b**^, *n* (%)								
Patient self-assesses	21 (15)	5 (9)	3 (5)	3 (15)	11 (28)	0 ^d^	2 (13)	0
Physician	22 (16)	8 (14)	2 (4)	1 (5)	9 (23)	4 (27)	5 (33)	2 (14)
Nurse	44 (32)	15 (26)	8 (15)	3 (15)	25 (63)	9 (60)	3 (20)	1 (7)
Dietitian/Nutritionist	98 (72)	43 (74)	52 (95)	18 (90)	27 (68)	10 (67)	9 (60)	10 (71)
Nutrition Assistant	7 (5)	4 (7)	4 (7)	3 (15)	1 (3)	1 (7)	1 (7)	0
Other	2 (1)	3 (5)	0	0	1 (3)	1 (7)	0	1 (7)
No response	21 (15)	8 (14)	3 (5)	0	0	1 (7)	4 (27)	3 (21)
**Anthropometrics measured as part of a nutritional assessment**^**b**^, *n* (%)								
Weight	115 (84)	50 (86)	54 (98)	18 (90)	33 (83)	14 (93)	14 (93)	13 (93)
Height	108 (79)	45 (78)	52 (95)	17 (85)	32 (80)	12 (80)	13 (87)	11 (79)
Body mass index	117 (85)	46 (79)	52 (95)	16 (80)^o^	39 (98)	13 (87)	14 (93)	12 (86)
Waist-to-hip ratio	5 (4)	4 (7)	2 (4)	1 (5)	2 (5)	1 (7)	1 (7)	1 (7)
Body surface area	13 (9)	9 (16)	2 (4)	1 (5)	7 (18)	2 (13)	2 (13)	4 (29)
Mid-arm muscle circumference	20 (15)	9 (16)	9 (16)	2 (10)	3 (8)	2 (13)	4 (27)	3 (21)
Skinfold thickness	15 (11)	2 (3)	9 (16)	0	2 (5)	0	3 (20)	1 (7)
Other	20 (15)	2 (3)^e^	11 (20)	1 (5)	7 (18)	1 (7)	1 (7)	0
None selected ^c^	13 (9)	6 (10)	0	1 (5)	1 (3)	1 (7)	0	0
**Body composition technology used as part of a nutritional assessment**^**b**^, *n* (%)								
Bioelectrical impedance analysis	26 (19)	7 (12)	10 (18)	1 (5)	8 (20)	3 (20)	4 (27)	1 (7)
Bioelectrical impedance spectroscopy	4 (3)	1 (2)	2 (4)	0	1 (3)	0	1 (7)	0
Computed tomography scan	11 (8)	7 (12)	3 (5)	0	6 (15)	2 (13)	0	3 (21)
Dual-energy X-ray absorptiometry	11 (8)	2 (3)	3 (5)	0	3 (8)	0	4 (27)	1 (7)
Ultrasound	5 (4)	0	1 (2)	0	1 (3)	0	1 (7)	0
Other	10 (7)	9 (16)^p^	3 (5)	2 (10)	6 (15)	3 (20)	1 (7)	1 (7)
None selected ^c^	87 (64)	37 (64)	38 (69)	17 (85)	19 (48)	8 (53)	9 (60)	9 (64)
**Pathology used as part of a nutritional assessment**^**b**^, *n* (%)								
Prealbumin	38 (28)	18 (31)	11 (20)	3 (15)	16 (40)	7 (47)	9 (60)	5 (36)
Transferrin	15 (11)	6 (10)	4 (7)	2 (10)	4 (10)	2 (13)	3 (20)	2 (14)
C-reactive protein	58 (42)	21 (36)	30 (55)	6 (30)^q^	14 (35)	8 (53)	7 (47)	4 (29)
Total lymphocyte count	26 (19)	11 (19)	5 (9)	3 (15)	14 (35)	6 (40)	1 (7)	2 (14)
Haemoglobin	45 (33)	20 (34)	19 (35)	7 (35)	15 (38)	7 (48)	2 (13)	4 (29)
Other	13 (9)	12 (21)^f^	4 (7)	3 (15)	7 (18)	4 (27)	0	3 (21)
None selected ^c^	55 (40)	24 (41)	20 (36)	12 (60)	15 (38)	4 (27)	4 (27)	4 (29)
**Functional assessments used as part of a nutritional assessment**^**b**^, *n* (%)								
WHO/ECOG/Zubrod Performance Status	35 (26)	16 (28)	4 (7)	5 (25)^r^	14 (35)	4 (27)	7 (47)	5 (36)
Karnofsky Performance Status	26 (19)	15 (26)	0	3 (15)^g^	12 (30)	4 (27)	9 (60)	7 (50)
Hand grip strength	34 (25)	12 (21)	14 (25)	1 (5)^s^	8 (20)	5 (33)	8 (53)	4 (29)
Other	7 (5)	2 (3)	2 (4)	1 (5)	4 (10)	0	0	1 (7)
None selected ^c^	14 (10)	6 (10)	37 (67)	14 (70)	14 (35)	6 (40)	2 (13)	4 (29)
**When nutritional assessment is conducted**^**b**^, *n* (%)								
At admission	32 (23)	11 (19)	6 (11)	6 (30)^h^	15 (38)	1 (7)^i^	4 (27)	2 (14)
When a patient is identified as at nutritional risk	97 (71)	45 (78)	53 (96)	15 (75)^j^	23 (58)	13 (87)^t^	13 (87)	14 (100)
On medical referral	53 (39)	22 (38)	35 (64)	6 (30)^k^	6 (15)	5 (33)	9 (60)	9 (64)
Other	17 (12)	3 (5)	12 (22)	0^l^	5 (13)	2 (13)	0	1 (7)
No response	14 (10)	6 (10)	0	0	0	0	0	0

*ECOG* Eastern Cooperative Oncology Group, *MNA* Mini Nutritional Assessment, *MUST* Malnutrition Universal Screening Tool, *PG-SGA* Patient-Generated Subjective Global Assessment, *SGA* Subjective Global Assessment, *WHO* World Health Organization

For single-response items, rounded percentages may have been adjusted by ± 1 percentage point to ensure a total of 100%, with adjustments based on proximity to rounding thresholds

^a^Due to the low number of participants from Asia reporting use of nutrition assessment in their workplace (*n* = 6 pre-COVID-19, *n* = 3 post-COVID-19), these data are not presented. Similarly, data from South America are not shown, since only two participants pre-COVID-19 were from South America, and none post-COVID-19

^b^Participants could provide more than one answer

^c^ ‘None selected’ indicates participants who did not select any of the listed methods. A ‘none of the above’ option was not provided

^d–l^Statistically significant differences between groups (pre-COVID-19 vs. post-COVID-19): ^d^*p* = 0.025 (Fisher’s exact test), ^e^*p* = 0.026 (Fisher’s exact test), ^f^*p* = 0.032 (Chi-square test), ^g^*p* = 0.017 (Fisher’s exact test), ^h^*p* = 0.046 (Chi-square test), ^i^*p* = 0.043 (Fisher’s exact test), ^j^*p* = 0.013 (Fisher’s exact test), ^k^*p* = 0.010 (Chi-square test), ^l^*p* = 0.029 (Fisher’s exact test)

^m–t^Trends indicating possible differences between groups (pre-COVID-19 vs. post-COVID-19) with *p*-values > 0.05 and ≤ 0.10): ^m^*p* = 0.096 (Fisher’s exact test), ^n^*p* = 0.081 (Fisher’s exact test), ^o^*p* = 0.077 (Fisher’s exact test), ^p^*p* = 0.077 (Chi-square test), ^q^*p* = 0.060 (Chi-square test), ^r^*p* = 0.051 (Fisher’s exact test), ^s^*p* = 0.057 (Fisher’s exact test), ^t^*p* = 0.058 (Fisher’s exact test)

Some notable shifts were observed in the specific methods used for nutritional assessment. While use of ‘other’ anthropometric methods decreased (from 15% to 3%, *p* = 0.026), the use of ‘other’ pathology-based methods increased significantly (from 9% to 21%, *p* = 0.032). Functional assessments remained variable, with the use of Karnofsky Performance Scale increasing significantly in Oceania (from 0% to 15%, *p* = 0.017).

Although nutritional assessment most frequently continued to occur post-COVID-19 when a patient was identified as being at nutritional risk, this practice significantly declined in Oceania following COVID-19 (from 96% to 75%, *p* = 0.013) as did assessments conducted following medical referral (from 64% to 30%, *p* = 0.010). Instead, the proportion of participants conducting nutrition assessment upon admission rose significantly (from 11% to 39%, *p* = 0.046). Furthermore, the use of “other” timing options declined to 0% (from 22%, *p* = 0.029). In Europe, assessments conducted at admission significantly decreased (from 38 to 7%, *p* = 0.043).

### Pre- and post-COVID-19 nutrition screening and assessment practices according to professional disciplines

Significant differences existed between nurses, dietitians, and physicians in their confidence in conducting nutrition screening both before and after COVID-19 (*p* < 0.001 for both) (Supplementary File 2). Before COVID-19, most dietitians (75%) reported very high confidence compared to only 28% of nurses and 13% of physicians. Post-COVID-19, this pattern largely persisted. Dietitians continued to report very high confidence at a similar rate (76%), whereas none of the nurses selected this response, and only 24% of physicians did. Instead, nurses and physicians were more likely to report average (44% and 41%) or high confidence (44% and 29%, respectively).

Dietitians predominantly reported taking less than 5 min to complete nutrition screening (82% pre-COVID-19, 82% post-COVID-19), whereas nurses and physicians before COVID-19 were split between requiring less than 5 min and requiring 5–10 min (*p* < 0.001 for comparison pre-COVID-19) (Supplementary File 2). After COVID-19, the proportion of nurses and physicians reporting taking less than 5 min to conduct a nutrition screening had increased (67% and 65%, respectively), and the difference between professional disciplines was no longer statistically significant (*p* = 0.246). For the estimated duration of conducting nutrition assessment, a significant difference between professional disciplines was observed pre-COVID, with fewer dietitians reporting that it took less than 10-min compared to nurses and physicians (*p* = 0.004). Post-COVID-19, this difference was no longer significant (*p* = 0.953), primarily due to a decrease in the proportion of nurses and physicians reporting durations under 10 min.

## Discussion

By comparing data from before the COVID-19 pandemic (2018) and after (2024), this study found some positive changes in how cancer care professionals identify protein-energy malnutrition (PEM) in their patients. This included an almost doubling of nutrition screening policies in North America and an improvement of routine nutrition screening at every outpatient appointment as well as nutrition assessments performed upon admission in Oceania. However, the repeated cross-sectional survey primarily identified that challenges in identifying PEM have either remained or have been exacerbated since the COVID-19 pandemic. Of significant concern, conducting nutritional assessments when indicated remained suboptimal and substantially declined in Oceania following identification of nutritional risk and upon medical referral post-COVID-19. While approximately half of participants reported that cachexia was assessed at their workplace, only 36% reported assessment of sarcopenia post-COVID-19, and 64% did not use body composition measures as part of their nutritional assessments. Despite considerable geographical variations, these findings may indicate a clinical focus on established wasting syndromes rather than on earlier, pre-cachectic stages. This poses a risk that PEM may not be recognised until it is overt, difficult to reverse, or advances to refractory cancer cachexia. Results underscore the need for efforts to facilitate implementation of assessment methods that not only include body weight but also consider body composition to enable early identification of PEM [16].

To our knowledge, no previous study has surveyed how nutritional care for patients with cancer has changed following COVID-19. Findings align with broader reports of disruptions to cancer care during the pandemic; however, as an observational study, no causal link can be established, and other factors may have influenced findings. In both pre- and post-COVID-19 surveys, the most perceived challenges to providing nutritional care were inconsistent follow-up, financial barriers, and health system–related barriers. While the survey did not further define health system–related barriers, and cultural differences between regions may lead to different perceptions of these, more specific barriers related to implementing nutrition screening were reported across time points, with the most pertinent being insufficient time, limited awareness, and insufficient staff. Notably, the proportion of participants reporting insufficient staff as a barrier increased significantly post-COVID-19 in parallel with a substantially lower proportion of participants reporting the hospital as a place of work than pre-COVID-19. These findings may reflect broader systemic strain, intensified by the pandemic, which may not have been adequately addressed in the post-COVID-19 era. Globally, staff shortages and widespread burnout have been well documented with many healthcare professionals since seeking work outside hospital settings [40–42]. Although there appears to have been an improved recognition of PEM as a priority globally, reflected by an increase in the number of workplaces that have nutrition screening policies (65% to 71%), the systemic strain may be undermining any implementation efforts and resulting in unchanged or worsened nutrition screening and assessment barriers and practices in most continents. Results also showed that while dietitians reported high confidence in conducting nutrition screening, confidence remained lower among physicians and nurses. As nurses are most frequently the ones responsible for screening in practice, this represents an additional barrier and underscores the need for targeted upskilling.

These findings highlight a need for practical approaches that can support nutrition screening and assessment implementation despite limited time and staffing resources. One such approach may be the use of digital screening systems, including electronic medical record–integrated prompts, app-based tools, or tablet-based intake registration, which can improve screening completion and reduce omissions by embedding nutrition screening into routine clinical workflows [43]. A notable finding, particularly alongside barriers such as insufficient time, insufficient staff, and low confidence in nutrition screening among nurses, was the post-COVID-19 increase in patient self-screening. This may reflect a movement towards patient empowerment to be more actively involved in self-management, as well as the accelerated adoption of telemedicine and patient-reported outcome measures following the pandemic [44–46]. The integration of nutrition screening into patient-completed tools could become a more widespread practice in the future, with a recent study having demonstrated that a patient-completed MST was feasible, acceptable, and showed greater accuracy than a clinician-completed MST [47]. Furthermore, patient-led screening may enhance patient awareness of nutrition, potentially facilitating earlier detection and prevention of PEM [48, 49]. The Head and Neck Cancer ‘ScreenIT’ study further supports this approach, demonstrating that patient-reported, system-driven screening processes can improve completion rates, timeliness of referral, and clinical outcomes. Where services already utilise patient-reported symptom monitoring systems, integrating nutrition screening into these platforms may represent a low-burden and scalable strategy to enhance early identification of PEM [50].

Conversely, self-completed nutrition assessments were observed to have the opposite trend, becoming an obsolete practice in most continents post-COVID-19, which may reflect the changes in the types of nutrition assessment tools utilised, some of which are not appropriate for self-assessment.

Collectively, these findings suggest that while the pandemic posed substantial barriers to nutritional care in cancer settings, it may also have catalysed important structural and digital developments, particularly regarding patient involvement in nutrition screening, that could advance early identification and management of PEM moving forward. Yet, the implementation of nutrition assessment for the diagnosis of PEM continues to face substantial challenges and has declined in some regions, with no compensatory practices detected.

While this study was not designed to generate formal practice recommendations, the findings provide a foundation for the MASCC Nutrition and Cachexia Study Group’s leadership to convene expert panels, review the emerging evidence, and lead a structured guideline-development process—such as through consensus workshops, Delphi rounds, or integration with existing national frameworks—to progress toward agreed, evidence-informed practice standards, including guidance on the use of electronic and patient-reported tools to support systematic early identification of PEM.

### Limitations

The lower post-COVID-2019 response rate may reflect a general survey fatigue in healthcare professionals following the pandemic [51]. Despite the international focus, the Asian, South American, and African continents were under-represented or not represented at all. A risk of sampling bias cannot be ruled out, since the survey was predominantly distributed through channels related to nutrition. Consequently, participants may have had a particular interest or expertise in nutrition, which could be associated with more optimal clinical practices. If this is the case, it is possible that our results may underestimate the challenges faced in the early identification of PEM, presenting a more favourable picture of practice than what might be found in less nutrition-aware settings.

The purpose-built questionnaire was developed by experienced clinicians as no validated instruments existed for assessing these specific nutrition care processes. However, the tool has not undergone formal psychometric validation, potentially limiting the generalisability of findings. A further limitation of this study is that implementation was not assessed against a recognised framework or using a tool mapped to such a framework, such as NoMAD [52]. Future research incorporating such measures is recommended to provide deeper insights into practice behaviours and implementation processes.

The high rate of non-responses on items related to nutritional assessment may be explained by a confusion of terminology. One participant reported feeling as though they had answered the same items twice, and it was determined that they did not distinguish between nutrition screening and nutritional assessment—a confusion that is well known in clinical practice [53]. This may have occurred for other participants but not reported to the research team. Better understanding of the concepts and consistent use of terminology may support improved interdisciplinary collaboration in the identification and treatment of PEM.

## Conclusion

Internationally, there have been substantial pre- and post-COVID-19 shifts to the perceptions and practices regarding PEM screening and assessment for patients with cancer among health professionals working in the cancer setting. While more workplaces have nutrition screening policies and there is a potentially beneficial improvement in patient-led nutrition screening, overall nutrition screening and nutrition assessment continue to face many barriers. This has led to a lack of improvement in nutrition screening and assessment practices in most regions and settings, and concerningly a decline in some cases such as nutrition screening awareness in North America or nutrition assessment implementation in Oceania. Nutrition screening and assessment practices must evolve for best patient care and in preparedness for future global health events.

## Supplementary Information

Below is the link to the electronic supplementary material.

ESM1 (DOCX 43.3 KB)

ESM2 (DOCX 22.6 KB)

## Data Availability

The datasets generated and/or analysed and questionnaire used during the current study are available from the corresponding author on reasonable request.
