# Supplementary material for: Impact of COVID-19 on early identification of protein-energy malnutrition in the cancer care setting: a repeated cross-sectional survey of cancer care professionals
Source: Support Care Cancer. 2026 Jan 22;34(2):117. doi: 10.1007/s00520-026-10338-1 (PMC12827316; doi:10.1007/s00520-026-10338-1)
Supplement: Supplementary file 1 — (DOCX 43.3 KB) [file 520_2026_10338_MOESM1_ESM.docx]

**Supplementary File 1: Sensitivity analyses adjusted for participants’ workplace(s)**

The categorisation of workplace responses is shown in Table S1a, while the results of both adjusted and unadjusted analyses of perceptions and practices regarding protein-energy malnutrition in the cancer setting are presented in Table S1b.

**Table S1a**: Categorisation of workplace responses for use in adjusted analyses

| **Category** | **Workplace**, n (%) | **Pre-COVID-19  (n=197)** | **Post-COVID-19  (n=85)** |
| --- | --- | --- | --- |
| **1** | ‘Hospital’ only | 78 (40) | 38 (45) |
| **2** | ‘Outpatient / Ambulatory’ (alone or combined with ‘University’ or ‘Homecare’) | 6 (3) | 12 (14) |
| **3** | ‘University’ (alone or combined with ‘Other’) | 11 (6) | 5 (6) |
| **4** | ‘Homecare’ (alone or combined with ‘Other’) | 2 (1) | 3 (4) |
| **5** | ‘Private practice’ | 1 (0) | 3 (4) |
| **6** | ‘Other’ (including ‘Hospice’) | 1 (0) | 3 (3) |
| **7** | ‘Hospital’ + ‘Outpatient / Ambulatory’ (including additional affiliation with ‘University’ or ‘Private practice’) | 30 (15) | 0 (0) |
| **8** | ‘Hospital’ + ‘University’ | 9 (5) | 1 (1) ^a^ |
| **9** | ‘Hospital’ + ‘Other’ (including Hospital’ + ‘Homecare’, ‘Hospital’ + ‘Private practice’ and additional affiliations to these combinations) | 7 (4) | 0 (0) |
| **10** | No response | 52 (26) | 20 (23) |

In the pre-COVID-19 survey, participants could select multiple answers to the workplace question. In the post-COVID-19 survey, only one answer was permitted. Rounded percentages may have been adjusted by ±1 percentage point to ensure a total of 100%, with adjustments based on proximity to rounding thresholds.
**a.** One participant in the post-COVID-19 survey indicated in a free-text response, that they worked in both ’Hospital’ and ‘University’.

**Table S1b**: Pre- and post-COVID-19 perceptions and practices of clinicians working in the cancer setting regarding protein-energy malnutrition in the cancer setting: Unadjusted and adjusted analyses

|  | **Unadjusted** | | **Adjusted** | |
| --- | --- | --- | --- | --- |
|  | Pre- COVID-19  (n=197) | Post- COVID-19 (n=85) | Pre- COVID-19  (n=197) | Post- COVID-19 (n=85) |
| **What “malnutrition” was perceived to mean in the clinical context**, % |  |  |  |  |
| Undernutrition | 60 | 68 | 60 | 68 |
| Overnutrition | 0 | 0 | 0 | 0 |
| Both undernutrition and overnutrition | 38 | 28 | 38 | 29 |
| Other | 1 | 3 | 1 | 2 |
| No response | 1 | 1 | 1 | 1 |
| **Perceived common nutritional challenges faced when caring for patients with cancer ^a^**, % |  |  |  |  |
| Inconsistent follow up | 48 | 46 | 49 | 43 |
| Health systems related barriers | 43 | 57 ^h^ | 43 | 57 ^i^ |
| Financial | 44 | 52 | 44 | 53 |
| Health Literacy ^b^ | 39 | 41 | 39 | 39 |
| Limited access to allied health | 28 | 31 | 29 | 29 |
| Cultural barriers ^b^ | 18 | 22 | 18 | 24 |
| Geographical considerations ^b^ | 17 | 16 | 16 | 18 |
| Language barriers ^b,c^ | 10 | 22 ^j^ | 10 | 25 ^k^ |
| Other ^b,d^ | 13 | 14 | 13 | 14 |
| **Malnutrition identification practices utilized in participants’ workplaces**, % |  |  |  |  |
| Nutrition screening | 9 | 14 | 10 | 11 |
| Nutrition assessment | 6 | 2 | 6 | 3 |
| Both nutrition screening and assessment | 64 | 66 | 63 | 67 |
| Neither | 4 | 7 | 4 | 7 |
| No response | 17 | 11 | 17 | 13 |
| **Sarcopenia assessed in participants workplace** ^b^, % |  |  |  |  |
| Yes | 41 | 36 | 40 | 38 |
| No | 49 | 52 | 49 | 53 |
| Unsure | 10 | 11 | 11 | 8 |
| No response | 0 | 1 | 0 | 1 |
| **Cachexia assessed in participants workplace** ^b^, % |  |  |  |  |
| Yes | 53 | 51 | 52 | 53 |
| No | 31 | 32 | 31 | 31 |
| Unsure | 11 | 9 | 12 | 7 |
| No response | 5 | 8 | 5 | 9 |
| **Existing nutrition screening policy at participants workplace** ^b^, % |  |  |  |  |
| Yes | 65 | 71 | 64 | 72 |
| No | 13 | 8 | 13 | 7 |
| Unsure | 5 | 6 | 6 | 4 |
| No response | 17 | 15 | 17 | 17 |
| **Perceived barriers to successful implementation of nutrition screening in participants workplace** ^e^, % |  |  |  |  |
| Insufficient time ^b^ | 46 | 48 | 47 | 46 |
| Limited awareness regarding the importance of nutrition screening ^b^ | 44 | 48 | 44 | 48 |
| Insufficient staff | 34 | 47 ^l^ | 33 | 49 ^m^ |
| Incorrect use of screening tool ^b^ | 28 | 21 | 28 | 21 |
| High patient turnover ^b^ | 26 | 21 | 26 | 24 |
| Unclear responsibility | 18 | 21 | 18 | 20 |
| Insufficient funding ^b,f^ | 9 | 14 | 12 | 20 |
| Social barriers ^g^ | 4 | 5 | 4 | 7 |

For single-response items, rounded percentages may have been adjusted by ±1 percentage point to ensure a total of 100%, based on proximity to rounding thresholds. In the adjusted analyses, participants’ workplace (categorized as shown in table S1a) was adjusted for using multinomial logistic regression for categorical outcomes and logistic regression for binary outcomes. Differences between pre- and post-COVID-19 surveys were assessed using either Chi-square or Fisher’s Exact test for unadjusted data and post-estimation Wald tests for adjusted data. The sample size (n) for adjusted analyses corresponds to the full study population; for variables where observations were excluded due to perfect prediction in the regression models, the effective sample size is indicated at the variable level with footnotes.
**a.** Participants could provide more than one answer. **b.** For the adjusted analyses of this variable, workplace categories 4, 5, and 6 were merged to avoid exclusions due to perfect prediction (binary variables) or convergence issues (categorical variables). **c.** Workplace category 3 (’University’, n=16) was excluded from the adjusted analysis due to perfect prediction, resulting in an adjusted sample size of n=266. The proportions of participants in this category were similar between pre-COVID-19 survey and post-COVID-19 survey, suggesting that the observed difference between surveys is unlikely to be affected by this exclusion. **d.** Workplace category 9 (’Hospital’+’Other’, n=7) was excluded from the adjusted analysis due to perfect prediction, resulting in an adjusted sample size of n=275. **e.** Participants were asked to indicate what they perceived as the top three most pertinent barriers. **f.** Workplace category 10 (’No response’, n=72) was excluded from the adjusted analysis due to perfect prediction, resulting in an adjusted sample size of n=210. **g.** Workplace categories 4, 5, 6, 8, and 9 (n=20) were excluded from the adjusted analysis due to perfect prediction, resulting in an adjusted sample size of n=252. **h–m**. Statistically significant differences between groups (pre-COVID-19 vs. post-COVID-19): **h.** p=0.040 (Chi-square test), **i.** p=0.048, **j.** p=0.006 (Chi-square test), **k.** p=0.006, **l.** p=0.031 (Chi-square test), **m.** p=0.012.
