# Supplementary material for: Impact of COVID-19 on early identification of protein-energy malnutrition in the cancer care setting: a repeated cross-sectional survey of cancer care professionals
Source: Support Care Cancer. 2026 Jan 22;34(2):117. doi: 10.1007/s00520-026-10338-1 (PMC12827316; doi:10.1007/s00520-026-10338-1)
Supplement: Supplementary file 2 — (DOCX 22.6 KB) [file 520_2026_10338_MOESM2_ESM.docx]

**Supplementary File 2**

**Table S2**: Pre- and post-COVID-19 nutrition screening and assessment practices according to professional disciplines among participants who reported using nutrition screening and/or assessment in their cancer care workplace

|  | **Pre-COVID-19** | | | | **Post-COVID-19** | | | | **Comparison between pre- and post-COVID-19  (p-value) ^b^** |
| --- | --- | --- | --- | --- | --- | --- | --- | --- | --- |
|  | **Dietitians (n=76)** | **Nurses (n=25)** | **Physicians**  **(n=23)** | **Difference between professional disciplines  (p-value) ^a^** | **Dietitians (n=33)** | **Nurses (n=9)** | **Physicians**  **(n=17)** | **Difference between professional disciplines  (p-value) ^a^** | *(The three professional disciplines combined)* |
| **Participants’ level of confidence in conducting a nutrition screening** ^c^, n (%) | | | | | | | | | |
| - Low confidence | 0 | 1 (4) | 1 (4) | <0.001 | 0 | 1 (11) | 1 (6) | <0.001 | 0.328 |
| - Average confidence | 5 (7) | 4 (16) | 7 (31) |  | 2 (6) | 4 (45) | 7 (41) |  |  |
| - High confidence | 14 (18) | 12 (48) | 12 (52) |  | 6 (18) | 4 (44) | 5 (29) |  |  |
| - Very high confidence | 57 (75) | 7 (28) | 3 (13) |  | 25 (76) | 0 | 4 (24) |  |  |
| - No response | 0 | 1 (4) | 0 |  | 0 | 0 | 0 |  |  |
| **Estimated duration for completing nutrition screening (as reported by participants)** ^c^, n (%) | | | | | | | | | |
| - Less than 5 minutes | 62 (83) | 12 (48) | 11 (48) | <0.001 | 27 (82) | 6 (67) | 11 (64) | 0.246 | 0.046 |
| - 5-10 minutes | 13 (17) | 12 (48) | 9 (39) |  | 5 (15) | 1 (11) | 3 (18) |  |  |
| - 10+ minutes | 0 | 1 (4) | 3 (13) |  | 1 (3) | 2 (22) | 3 (18) |  |  |
| **Estimated duration for completing nutrition assessment (as reported by participants)** ^d^, n (%) | | | | | | | | | |
|  | (n=73) | (n=21) | (n=25) | 0.004 | (n=31) | (n=6) | (n=14) | 0.953 | 0.431 |
| - Less than 10 minutes | 19 (26) | 12 (57) | 14 (56) |  | 9 (29) | 2 (33) | 4 (28) |  |  |
| - 10-20 minutes | 30 (41) | 8 (38) | 6 (24) |  | 11 (36) | 3 (50) | 5 (36) |  |  |
| - 20+ minutes | 24 (33) | 1 (5) | 4 (16) |  | 11 (35) | 1 (17) | 5 (36) |  |  |
| - No response | 0 | 0 | 1 (4) |  | 0 | 0 | 0 |  |  |

Rounded percentages may have been adjusted by ±1 percentage point to ensure a total of 100%, with adjustments based on proximity to rounding thresholds.
**a.** Differences between groups are tested using Fisher’s Exact test. **b.** Differences between groups are tested using Fisher’s Exact test for questions on nutrition screening and Chi-square test for question on nutrition assessment. **c.** Only participants who reported using nutrition screening in their workplace were included. **d.** Only participants who reported using nutrition assessment in their workplace were included.
